# Supplementary material for: Indole and 2,4-Thiazolidinedione conjugates as potential anticancer modulators
Source: PeerJ. 2018 Aug 8;6:e5386. doi: 10.7717/peerj.5386 (PMC6087425; doi:10.7717/peerj.5386)
Supplement: Appendix 1 — Spectral data of compounds and Table S1. [file peerj-06-5386-s001.docx]

**Indole and 2,4-Thiazolidinedione conjugates as potential anticancer modulators**

Domenica M. Corigliano^1,5^, Riyaz Syed^2,5^, Sebastiano Messineo^1^, Antonio Lupia^1^, Rahul Patel^3^, Chittireddy V.R. Reddy^2^, Pramod K. Dubey^2^, Carmen Colica^4^, Rosario Amato^1^, Giovambattista De Sarro^1^, Stefano Alcaro^1^, Adisherla Indrasena^2,*^ and Antonio Brunetti^1,*^

^1^ Department of Health Sciences, University “Magna Græcia” of Catanzaro, Catanzaro, Italy

^2^ Department of Chemistry, Jawaharlal Nehru Technological University Hyderabad, Kukatpally, Hyderabad, India

^3^ Dongguk University‐Seoul, Department of Food Science and Biotechnology, Ilsandong‐gu,

Goyang‐si, Gyeonggi‐do, Republic of Korea.

^4^ CNR, IBFM UOS of Germaneto, University “Magna Graecia” of Catanzaro, Catanzaro, Italy

Supplementary Appendix

**SPECTRAL DATA OF COMPOUNDS**

**AC1** IR (KBr): 3205, 3029 (-NH groups), 1725, 1660 cm^-1^ (-C=O groups); ^1^H NMR (400 MHz, DMSO-*d_6_*_/_TMS): δ 7.43-8.61 (m, 6H, 4H aromatic + 1H α-indolyl + 1H vinylic proton), 12.11 (s, H, -NH, D_2_O exch), 12.48 (s, H, -NH, D_2_O exch.); ^13^C NMR (100 MHz, DMSO-*d_6_*/TMS): 111.15, 113.56, 119.13, 120.34, 123.34, 124.72, 125.61, 126.55, 130.43, 137.87,167.70, 173.88; m/z: 243.6 [M-1]^+^.

**AC2** IR (KBr): 3036 (-NH group), 1718, 1667 cm^-1^ (-C=O groups); ^1^H NMR (400 MHz, DMSO-*d_6_*_/_TMS): δ 7.25-8.91 (m, 11H, 4H aromatic + 5H aromatic + 1H α-indolyl + 1H vinylic proton), 12.24 (s, H, -NH, D_2_O exch); ^13^C NMR (100 MHz, DMSO-*d_6_*/TMS):103.12, 111.26, 112.41, 112.67, 113.43, 115.72, 117.38, 119.08, 120.41, 123.46, 124.60, 125.54, 126.09, 126.47, 130.30, 137.85,167.69, 173.84; m/z: 383.4 [M-1]^+^.

**AC3** IR (KBr): 1725, 1672 cm^-1^ (-C=O groups); ^1^H NMR (400 MHz, DMSO-*d_6_*_/_TMS): δ 2.94 (s, 2H, -NCH_2_ of TZD), 3.65 (s, 3H, -NCH_3_ of indole), 7.20-8.74 (m, 11H, 4H aromatic + 5H aromatic + 1H α-indolyl + 1H vinylic proton); ^13^C NMR (100 MHz, DMSO-*d_6_*/TMS): 30.12, 33.42, 102.15, 107.46, 112.58, 113.40, 113.79, 115.65, 117.23, 119.15, 120.54, 123.35, 124.66, 125.34, 126.41, 130.27, 137.76,167.54, 173.91; m/z: 347.2 [M-1]^+^.

**AC4** IR (KBr): 3204, 3021 (-NH groups), 1724, 1676 cm^-1^ (-C=O groups); ^1^H NMR (400 MHz, DMSO-*d_6_*_/_TMS): δ 7.57-8.57 (m, 5H, 3H aromatic + 1H α-indolyl + 1H vinylic proton), 12.26 (s, H, -NH, D_2_O exch), 12.53 (s, H, -NH, D_2_O exch.); ^13^C NMR (100 MHz, DMSO-*d_6_*/TMS): 103.02, 111.26, 113.62, 119.03, 120.21, 123.15, 124.61, 125.68, 126.64, 130.31, 137.91,167.75, 173.98; m/z: 268.7 [M-1]^+^.

**AC5** IR (KBr): 3208, 3026 (-NH groups), 1719, 1683 cm^-1^ (-C=O groups); ^1^H NMR (400 MHz, DMSO-*d_6_*_/_TMS): δ 7.06-8.05 (m, 5H, 3H aromatic + 1H α-indolyl + 1H vinylic proton), 12.19 (s, H, -NH, D_2_O exch), 12.30 (s, H, -NH, D_2_O exch.); ^13^C NMR (100 MHz, DMSO-*d_6_*/TMS): 110.45, 112.37, 116.60, 118.30, 120.97, 123.00, 124.14, 126.76, 128.52, 136.18, 167.75, 167.90; m/z: 261 [M-1]^+^.

**AC6** IR (KBr): 1728, 1674 cm^-1^ (-C=O groups); ^1^H NMR (400 MHz, DMSO-*d_6_*_/_TMS): δ 3.16 (s, 3H, -NCH_3_of TZD), 3.60 (s, 3H, -NCH_3_ of indole), 6.22-8.85 (m, 5H, 3H aromatic + 1H α-indolyl + 1H vinylic proton); ^13^C NMR (100 MHz, DMSO-*d_6_*/TMS): 30.08, 34.23, 109.82, 114.32, 115.01, 116.23, 122.21, 129.00, 130.98, 131.91, 145.08, 158.46, 168.23, 169.81; m/z: 291 [M+1]^+^.

**AC7** IR (KBr): 1731, 1675 cm^-1^ (-C=O groups); ^1^H NMR (400 MHz, DMSO-*d_6_*_/_TMS): δ 1.15-1.18 (t, 3H, -CH_3_), 1.37-1.41 (t, 3H, -CH_3_), 3.65-3.71 (q, 2H,-CH_2_-), 4.36-4.39 (q, 2H,-CH_2_-), 7.13-8.14 (m, 5H, 3H aromatic + 1H α-indolyl + 1H vinylic proton); ^13^C NMR (100 MHz, DMSO-*d_6_*/TMS): 12.85, 15.20, 36.37, 41.53, 109.75, 109.79, 111.16, 111.42, 112.13, 112.22, 114.04, 124.92, 128.09, 128.20, 132.33, 165.25, 166.84; m/z: 319 [M+1]^+^.

**AC8** IR (KBr): 1745, 1670 cm^-1^ (-C=O groups); ^1^H NMR (400 MHz, DMSO-*d_6_*_/_TMS): δ 3.49-3.67 (s, 2H,-CH_2_-), 4.30-4.41 (s, 2H,-CH_2_-), 7.10-8.79 (m, 15H, 3H aromatic of indole + 10H aromatic of aryl groups + 1H α-indolyl + 1H vinylic proton); ^13^C NMR (100 MHz, DMSO-*d_6_*/TMS): 12.65, 15.34, 45.64, 109.68, 109.84, 111.23, 111.39, 112.21, 112.35, 114.11, 122,00, 124.87, 125.70, 126.80, 126.90, 126.92, 127.58, 127.01, 128.15, 128.16, 128.23, 128. 30, 128.31, 132.42, 165.29, 166.76; m/z: 442.1 [M+1]^+^.

**AC9** IR (KBr): 3208, 3026 (-NH groups), 1719, 1683 cm^-1^ (-C=O groups); ^1^H NMR (400 MHz, DMSO-*d_6_*_/_TMS): δ 7.22-8.28 (m, 5H, 3H aromatic + 1H α-indolyl + 1H vinylic proton), δ 12.15 (s, H, -NH, D_2_O exch), 12.32 (s, H, -NH, D_2_O exch); ^13^C NMR (100 MHz, DMSO-*d_6_*/TMS): 110.36, 112.28, 116.58, 118.51, 120.75, 123.12, 124.23, 126.63, 128.46, 136.22, 167.64, 167.88; m/z: 278 [M]^+^, 280 [M+2] ^+^.

**AC10** IR (KBr): 1730, 1655 cm-^1^ (-C=O groups); ^1^H NMR (400 MHz, DMSO-*d_6_*_/_TMS): δ 3.16 (s, 3H, -NCH_3_of TZD), 3.60 (s, 3H, -NCH_3_ of indole), 6.22-8.85 (m, 5H, 3H aromatic + 1H α-indolyl + 1H vinylic proton); ^13^C NMR (100 MHz, DMSO-*d_6_*/TMS): 31.98, 33.34, 111.23, 111,98, 113.72, 122.09, 123.82, 124.38, 128.23, 131.75, 135.53, 145.73, 167.02, 170.19; m/z: 351 [M]^+^, 353 [M+2]^+^.

**AC11** IR (KBr): 3520 cm^-1^ (-NH), 1751, 1640 cm^-1^(-C=O groups); ^1^H NMR (400 MHz, DMSO-*d_6_*/TMS): δ 2.46 (s, 3H, -CH_3_), δ 7.09-7.86 (m, 5H, 3H aromatic + 1H α-indolyl + 1H vinylic proton), δ 12.43 (s, 1H, -NH, D_2_O exch); ^13^C NMR (100 MHz, DMSO-*d_6_*/TMS): 33.70, 111.46, 111.71, 116.11, 118.14, 118.76, 122.83, 126.41, 135.13, 139.54, 142.10, 167.15, 167.35; m/z: 337.19 [M+1]^+^.

**AC12** IR (KBr): 3208, 3021 (-NH groups), 1714, 1676 cm^-1^ (-C=O groups); ^1^H NMR (400 MHz, DMSO-*d_6_*_/_TMS): δ 7.01-8.12 (m, 5H, 3H aromatic + 1H α-indolyl + 1H vinylic proton), 12.25 (s, H, -NH, D_2_O exch), 12.42 (s, H, -NH, D_2_O exch.); ^13^C NMR (100 MHz, DMSO-*d_6_*/TMS): 110.36, 112.37, 116.54, 118.36, 120.85, 123.13, 124.23, 126.64, 128.50, 136.21, 167.81, 167.94; m/z: 321.3 [M-1]^+^.

**AC13** IR (KBr): 3212, 3032 (-NH groups), 1718, 1664 cm^-1^ (-C=O groups); ^1^H NMR (400 MHz, DMSO-*d_6_*_/_TMS): δ 7.37-8.58 (m, 5H, 3H aromatic + 1H α-indolyl + 1H vinylic proton), 12.19 (s, H, -NH, D_2_O exch), 12.37 (s, H, -NH, D_2_O exch.); ^13^C NMR (100 MHz, DMSO-*d_6_*/TMS):112.46, 112.85, 115.72, 118.05, 118.84, 123.08, 125.29, 128.88, 131.33, 139.18, 141.89 167.18, 167.48; m/z: 289.3 [M]^+^.

**AC14** IR (KBr): 3226 (-NH group), 1722, 1653 cm^-1^ (-C=O groups); ^1^H NMR (400 MHz, DMSO-*d_6_*_/_TMS): δ 2.98 (s, 3H, N-CH_3_), 7.41-8.63 (m, 5H, 3H aromatic + 1H α-indolyl + 1H vinylic proton), 12.32 (s, H, -NH, D_2_O exch); ^13^C NMR (100 MHz, DMSO-*d_6_*/TMS): 33.72, 111.52, 111.63, 116.06, 118.06, 118.83, 122.90, 126.49, 135.02, 139.51, 142.16, 167.10,167.46; m/z: 303.2 [M]^+^.

**AC15** IR (KBr): 3231 (-NH group), 1729, 1648 cm^-1^ (-C=O groups); ^1^H NMR (400 MHz, DMSO-*d_6_*_/_TMS): δ 1.56-1.97 (t, 3H, -CH_3_), 3.45-3.85 (q, 2H,-CH_2_-), 7.34-8.71 (m, 5H, 3H aromatic + 1H α-indolyl + 1H vinylic proton), 12.29 (s, H, -NH, D_2_O exch); ^13^C NMR (100 MHz, DMSO-*d_6_*/TMS):15.23, 41.75, 111.40, 111.98, 116.06, 117.99, 119.52, 122.31, 126.63, 133.41, 138.46, 142.02, 167.11,167.35; m/z: 317.3 [M]^+^.

**AC16** IR (KBr): 1730, 1656 cm^-1^ (-C=O groups); ^1^H NMR (400 MHz, DMSO-*d_6_*_/_TMS): δ 1.09-1.15 (t, 3H, -CH_3_), 1.32-1.39 (t, 3H, -CH_3_), 3.59-3.65 (q, 2H,-CH_2_-), 4.15-4.43 (q, 2H,-CH_2_-), 7.29-8.65 (m, 5H, 3H aromatic + 1H α-indolyl + 1H vinylic proton); ^13^C NMR (100 MHz, DMSO-*d_6_*/TMS): 12.3, 15.31, 41.68, 111.66, 111.94, 116.11, 117.85, 119.50, 122.42, 126.68, 127.5, 133.35, 138.39, 142.13, 167.26,167.68; m/z: 345.3 [M]^+^.

**AC17** IR (KBr): 1716, 1639 cm^-1^ (-C=O groups); ^1^H NMR (400 MHz, DMSO-*d_6_*_/_TMS): δ 3.10 (s, 3H, -NCH_3_of TZD), 3.57 (s, 3H, -NCH_3_ of indole), 7.12-8.69 (m, 5H, 3H aromatic + 1H α-indolyl + 1H vinylic proton); ^13^C NMR (100 MHz, DMSO-*d_6_*/TMS): 31.85, 33.26, 111.34, 111,91, 113.60, 122.16, 123.75, 124.41, 128.20, 131.82, 135.49, 145.66, 167.12, 167.56; m/z: 317.0 [M]^+^.

**AC18** IR (KBr): 3342, 3265 cm^-1^ (-NH groups), 1710, 1676 cm^-1^ (-C=O groups); ^1^H NMR (400 MHz, DMSO-d_6/_TMS): δ 3.82 (s, 3H, -OCH_3_), 6.84-6.09 (m, 5H, 3H aromatic + 1H α-indolyl + 1H vinylic proton), δ 12.00 (s, H, -NH, D_2_O exch.), 12.25 (s, H, -NH, D_2_O exch); ^13^C NMR (100 MHz, DMSO-d_6_/TMS): 55.82, 104.83, 110.23, 112.24, 112.51, 123.09, 128.34, 128.42, 129.47, 144.28, 154.23, 166.76, 168.22; m/z: 275 [M+1]^+^.

**AC19** IR (KBr): 1710, 1684 cm^-1^ (-C=O groups); ^1^H NMR (400 MHz, DMSO-*d_6_*_/_TMS): δ 3.14 (s, 3H, -NCH_3_of TZD), 3.58 (s, 3H, -NCH_3_ of indole), 3.78 (s, 3H, -OCH_3_), 6.28-8.79 (m, 5H, 3H aromatic + 1H α-indolyl + 1H vinylic proton);  ^13^C NMR (100 MHz, DMSO-*d_6_*/TMS): 33.72, 35.85, 58.34, 111.52, 111.63, 116.06, 118.06, 118.83, 122.90, 126.49, 135.02, 139.51, 142.16, 167.10, 167.46; m/z: 303 [M+1]^+^.

**AC20** IR (KBr): 1723, 1675 cm^-1^ (-C=O groups); ^1^H NMR (400 MHz, DMSO-*d_6_*_/_TMS): δ 1.05-1.19 (t, 3H, -CH_3_), 1.45-1.52 (t, 3H, -CH_3_), 3.41-3.60 (q, 2H,-CH_2_-), 4.10-4.38 (q, 2H,-CH_2_-), 3.63 (s, 3H, -OCH_3_), 6.92-8.80 (m, 5H, 3H aromatic + 1H α-indolyl + 1H vinylic proton); ^13^C NMR (100 MHz, DMSO-*d_6_*/TMS): 12.3, 15.1, 33.56, 35.73, 58.30, 111.44, 111.51, 116.13, 118.15, 118.70, 122.86, 126.57, 135.11, 139.43, 142.77, 167.05, 167.33; m/z: 331 [M+1]^+^.

**AC21** mp. > 250 °C; yield: 0.22 gr (90%); IR (KBr): 3197, 3150 (-NH), 1741, 1673, 1613 cm^-1^ (-C=O); ^1^H NMR (400 MHz, DMSO-*d_6_*/TMS): δ 6.93-8.77 (m, 4H, aromatic), 11.20 (s, -NH, D_2_O exch.), 12.79 (s, -NH, D_2_O exch.); ^13^C NMR (100 MHz, DMSO-*d_6_*/TMS): 61.97, 75.71, 109.32, 121.07, 124.62, 129.23, 129.81, 142.72, 177.38, 181.15, 188.05; m/z: 247 [M+1]^+^.

**AC22** mp. > 250 °C; yield: 0.23 gr (88%); IR (KBr): 3177, 3138 (-NH), 1756, 1683, 1623 cm^-1^ (-C=O); ^1^H NMR (400 MHz, DMSO-*d_6_*/TMS): δ 6.90-8.55 (m, 3H, aromatic), 11.19 (s, -NH, D_2_O exch.), 12.81 (s, -NH, D_2_O exch.); ^13^C NMR (100 MHz, DMSO-*d_6_*/TMS): 61.82, 75.65, 109.41, 121.10, 124.68, 129.37, 129.76, 142.69, 177.23, 181.11, 188.10; m/z: 265 [M+1]^+^.

**AC23** mp. > 250 °C; yield: 0.24 gr (85%); IR (KBr): 3165, 3135 (-NH), 1765, 1653, 1608 cm^-1^ (-C=O); ^1^H NMR (400 MHz, DMSO-*d_6_*/TMS): δ 6.89-8.62 (m, 3H, aromatic), 11.09 (s, -NH, D_2_O exch.), 12.75 (s, -NH, D_2_O exch.); ^13^C NMR (100 MHz, DMSO-*d_6_*/TMS): 61.85, 75.63, 109.20, 121.12, 124.75, 129.29, 129.77, 142.64, 177.42, 181.09, 188.15; m/z: 280 [M]^+^, 282 [M+2]^+^.

**AC24** mp. > 250 °C; yield: 0.28 gr (87%); IR (KBr): 3153, 3148 (-NH), 1750, 1643, 1618 cm^-1^ (-C=O); ^1^H NMR (400 MHz, DMSO-*d_6_*/TMS): δ 6.88-8.71 (m, 3H, aromatic), 11.17 (s, -NH, D_2_O exch.), 12.82 (s, -NH, D_2_O exch.); ^13^C NMR (100 MHz, DMSO-*d_6_*/TMS): 61.80, 75.54, 109.18, 121.08, 124.67, 129.35, 129.62, 142.35, 177.62, 181.24, 188.36; m/z: 325 [M]^+^, 327 [M+2]^+^.

**AC25** mp. > 250 °C; yield: 0.33 gr (90%); IR (KBr): 3183, 3148 (-NH), 1772, 1657, 1623 cm^-1^ (-C=O); ^1^H NMR (400 MHz, DMSO-*d_6_*/TMS): δ 6.79-8.73 (m, 3H, aromatic), 11.08 (s, -NH, D_2_O exch.), 12.79 (s, -NH, D_2_O exch.); ^13^C NMR (100 MHz, DMSO-*d_6_*/TMS): 61.74, 75.62, 109.09, 121.11, 124.53, 129.39, 129.62, 142.30, 177.54, 181.34, 188.47; m/z: 373 [M+1]^+^.

**AC26** mp. > 250 °C; yield: 0.22 gr (83%); IR (KBr): 3175, 3129 (-NH), 1764, 1692, 1634 cm^-1^ (-C=O); ^1^H NMR (400 MHz, DMSO-*d_6_*/TMS): δ 7.02-8.64 (m, 3H, aromatic), 11.73 (s, -NH, D_2_O exch.), 12.87 (s, -NH, D_2_O exch.); ^13^C NMR (100 MHz, DMSO-*d_6_*/TMS): 61.64, 75.52, 109.15, 121.34, 124.49, 129.28, 129.50, 142.41, 177.46, 181.28, 188.56; m/z: 265 [M+1]^+^.

**Table S1**: Glide and Induced Fit Docking scores of rosiglitazone and AC18, AC20 and AC22 best poses in PPARγ pocket.

| **Compounds** | **Glide SP** | **Induced** | **Fit** |
| --- | --- | --- | --- |
|  | Glide G-score* | Glide G-score* | IFD Score* |
| Rosigltazone | -11.157 | -11.404 | -534.229 |
| AC18 | -8.262 | -10.643 | -973.494 |
| AC20 | -8.476 | -10.760 | -973.821 |
| AC22 | -8.507 | -9.708 | -967.612 |

*In kcal/mol.
